# A Visit to Foreign Dental Schools and Other Observations

**Published:** 1886-07

**Authors:** A. W. Harlan

**Affiliations:** Chicago, Illinois.


ARTICLE III.

### A VISIT TO FOREIGN DENTAL SCHOOLS AND OTHER OBSERVATIONS.

BY A. W. HARLAN, M. D., D. D. S., CHICAGO, ILLINOIS.

A recent visit to Europe enabled me to observe the workings of the dental schools of London, Berlin and Paris. Before describing what I saw and heard in London, a few preliminary remarks concerning requirements for admission to English dental hospitals may be useful. Applicants for entrance to British dental schools, who commenced the study of dentistry prior to 1878, are not required to pass the entrance examinations; all others must undergo a preliminary entrance examination, comprising English language, grammar and composition, English history, modern geography, Latin, including grammar and translation, elements of mathematics, vulgar and decimal fractions, algebra (simple equations), geometry, including the first two books of Euclid, elementary mechanics of solids and fluids, including statics, dynamics and hydrostatics, and one of the following optional subjects: Greek, French, German, Italian or other modern language, logic, botany or elementary chemistry.

When the student has fulfilled the above requirements he is required to register himself as a dental student at the office of the General Medical Council. After such registration he must pursue his studies for four years in one of the recognized schools, including in that period an apprenticeship in mechanical dentistry under some registered dentist. Before taking his final examination for the L. D. S. degree, he must attain the age of twenty-one years. During the four years of studentship he attends lectures on general anatomy, pathology, chemistry, surgery, materia medica, physiology, and other general medical and scientific subjects in a regular medical school. He also does his dissecting, chemical and histological work, including the work of dresser or assistant in a hospital ward in the same school. Dental anatomy, physiology, surgery, mechanical and operative dentistry, special therapeutics, anæsthesia and other special subjects, are taught in the dental hospital, including practical work in operative dentistry.

Instructions in mechanical dentistry, as before mentioned, is obtained from private sources. The theory of mechanical dentistry, including carving of bone, ivory, etc., manufacture of instruments, swaging, soldering, and the putting up of specimen cases, is taught in the dental hospital. Practical cases are not made in the dental schools of London. (I was so informed.)

On entering the Dental Hospital of London, (founded 1859), situated on one side of Leicester Square, you at first find yourself in the reception room for patients (which is open daily, except Sundays, from 9 to 11 A. M.) A clerk or book-keeper records the age, sex, residence, occupation and other facts of this nature relating to the patient, including the kind of operation which is required for his relief,

(filling, extracting, correction of irregularity, cleansing teeth, surgical operation, or other required service.) The patient then goes up stairs, where he is received by the house surgeon or his assistant, by whom he is assigned to the student. There are always plenty of patients. If an anæsthetic is to be administered it is given by the regularly appointed anæsthetist of the school, or under his direction. He attends daily. At least one clinical instructor is present daily, who performs some operation in filling or otherwise, during his hours of service. The house surgeon and his assistant have charge of the operating rooms, and furnish the materials for filling, etc., to the student, who collects the fee. When the student gets a sheet of gold (No. 4) he pays thirty-six cents for it, and of course gets as much or more from the patient. No charges are made for plastic fillings, tin, gutta-percha, or other services, except for gold, as above stated. This has a tendency to discourage the use of gold by the patient. He prefers the filling which costs nothing. The student, in consequence, does not get from this method of fees as much practical use of gold, even in twice the length of time, as he obtains in an American dental college. From what I saw I should say that very little cohesive gold is used by students in the hospital. Certainly not many large and complicated gold fillings are made by them during the two years' clinical work. They obtain a knowledge of the use of non-cohesive gold, however, which is perhaps quite as valuable in practice, because the English dentists as a class (with few exceptions) do not make, or attempt to make, large gold fillings, preferring plastics, pivoting or extraction, when cavities are large or teeth are pulpless: as they argue, from the system of fees which are in vogue, that it does not pay the operator; that people will not submit to prolonged operations, and that in many cases large gold fillings will not prove as serviceable (through lack of care of the teeth after filling, etc.) as frequently-renewed plastic fillings.

Root filling is taught, but I fear many (at present) do not practice it with that degree of care and thoroughness which we deem essential to success. It is not considered good practice in America, I believe, to fill roots of teeth with cotton, or to leave them unfilled and drill a vent-hole in the side of the root. Many dentists in Great Britain and on the continent practice in this way daily. American methods of filling teeth, and roots of teeth, have not taken that deep hold on the European practitioner which some theorists would gladly have one believe. Many foreign dentists—like some at home—read nearly everything that is published, but do not put into practice what in many cases would be better for their clients. They are content with the knowledge they possess, and do not easily or readily take up with new ideas. They are too conservative.

The rubber dam is used in the hospital. The gentlemanly house surgeon explained the methods of teaching, and was at considerable pains to show the *modus operandi* of ordinary operations. I think they have about one chair (not modern) for every three or four students. The operating rooms, although located on the fourth floor, are not well lighted, and are not sufficiently commodious, as there are two or three rows of chairs back from the windows. Dental engines were numerous, and many of them were in actual use. The students are not boisterous, they indulged in no loud talking, and appeared to be somewhat older than the average dental student at home.

Located in the same building is the office of the British Dental Association, and the journal of that society is issued from thence. The Odontological Society of Great Britain is also located on the lower floors, and their museum, rich in models, casts, skulls and other valuable materials in human and comparative anatomy, is open to the student desirous of gathering knowledge. The past and present students have a society, which holds monthly meetings in the hospital, an exceedingly great advantage for the juniors. They hold annual reunions and give a dinner to encourage social intercourse. Outside the entrance is a box for contributions for the support of the hospital. Soirees and subscription parties are also given from time to time for the support of the hospital. I thought, in ruminating over the subject, that if small fees were collected for all plastic filling operations, the contributions which are made by the benevolent, and the other funds coming into the hospital, might be used to reduce the cost of operations in gold, and thereby benefit the student by teaching him from actual practice the better methods of operating. I do not wish to be misunderstood in the above paragraph. The student is taught the methods, but he does not have enough practice in the use of gold while he is a student. The British journals publish a list of the operations performed in the various hospitals every month, and any one can see the justice of these remarks. Here is one of the late reports:

*Monthly Report of cases treated at the Dental Hospital of London, from October 1st to October 31st, 1885.*

| | | |
|---|---|---|
| Extractions— | Children under 14 | 378 |
| | Adults | 912 |
| | Under Nitrous Oxide | 276 |
| Gold Stoppings | | 267 |
| Other Stoppings | | 879 |
| Advice | | 121 |
| Irregularities of the Teeth | | 97 |
| Miscellaneous Cases | | 387 |

*National Dental Hospital—same month.*

| | | |
|---|---|---|
| Extractions— | Children under 14 | 424 |
| | Adults | 555 |
| | Under Nitrous Oxide | 614 |
| Gold Stoppings | | 121 |
| Other Stoppings | | 625 |
| Advice and Scaling | | 421 |
| Irregularities of the Teeth | | 409 |
| Miscellaneous Cases | | 146 |

Each statement is signed by the respective house surgeon. No report of roots filled, or abscesses treated, or crowns or pivot teeth adjusted. The records speak for themselves. In the report of the National Dental Hospital, for the year 1885, there is a record of 9,001 fillings, of which number 1,014 were made with gold. I have not seen the report of the Dental Hospital of London for the same year, but the monthly reports of fillings average about the same; that is to say, not quite twelve gold fillings in every hundred inserted. One unconsciously gathers from this, that the insertion of such a large percentage of fillings other than gold has a tendency to discourage thorough cleansing and preparation of cavities. Hence the frequent failure of plastic operations.

I visited the National Dental Hospital also, and the methods of teaching are substantially the same, the hours of attendance of patients, operators, house surgeons and clinical instructors, occupying about the same number of hours. This school is younger and it occupies smaller quarters, but in other respects I should judge that the instruction is quite as thorough and scientific as that given in the older school. The fees are not quite as high. I found the house surgeon quite as willing to show me the working of the school as his *confrere* in Leicester Square. I visited the hospital on a rainy morning, in the company of another American dentist, and while there a discussion arose concerning the use of filling materials. The house surgeon argued that it was almost useless to insert gold fillings for the class of patients who visit infirmaries, as such people took no care of their teeth. I took the other side, or the student's side, which was that it was a benefit to him, as it taught the use of instruments, the manipulation of gold, and that he would be better prepared to operate for himself when launched into the arena of daily personal practice. The question was not settled, but I hope that I impressed him with the importance of the proposition. This is the principal observable defect in the clinical instruction in each school. If there are forty students in a school for the year, and only 1,000 fillings of gold inserted during that time, it indicates a small average in the total number of fillings for each student.

The English student is well instructed in the use of anæsthetics; much better than are Americans. He learns more of comparative anatomy than we teach, and generally well drilled in normal and pathological histology. Dental surgery and special therapeutics, I believe from what I saw and heard, are better understood at home, by our college-educated dentists, than by our English cousins. This is my impression from many conversations held with dentists of low and high degree. They are better mechanics in the work-shop, *en masse*, but not so ingenious or inventive. When it comes to the final examination, we must take a back seat, as the licensing bodies are not the teaching corps. When we adopt—as we must in time, and I hope very soon—that feature of professional education, then will our diplomas be like Cæsar's wife, above suspicion.

We deliver more didactic lectures in a six month's course in America than an English student listens to in eighteen months. By different methods we arrive at the same result. They consume more time, but place them side by side in practice in a working society, in the field of journalistic contributors, and our own American graduates will hold their ground quite as well as the subjects of the Queen. The amount of valuable material published in professional journals in America attests this.

The British dentist is more social, and that element in his nature almost overshadows the scientific and practical side, even in dental societies. Their method of conducting meetings of societies has much in it to commend. Members do not straggle in at all hours, after business has begun, and no talking or whispering goes on while a speaker has the floor. The business of the meeting is conducted in a dignified manner. This to some might appear dull and prosy, but it pleased me. Scientific work is no laughing matter, and for a few boisterous, ill-mannered persons to talk and laugh and whisper while a scientific paper is being read, which has required weeks or months of labor to prepare, is a poor compliment to pay to the author. Hence his decorousness impressed me more forcibly, as I have been in society meetings where attention was almost wholly diverted from the business in hand, to listen to a story or other trivial matter.

English fees are not based on anything but tradition. There is no justice to the operator in receiving but a guinea for his maximum fee. I will not say that larger fees are not charged or collected by English dentists, but the custom for those of the highest rank is to receive about $5.00 for each operation performed, be it easy or laborious. Americans practicing in Great Britain usually try to transplant American ideas, but they do not all succeed, as I heard of some who have adopted the English custom. Fees for artificial teeth are even higher than in America—and also lower—for in America no one ever heard of a dentist inserting a single tooth on rubber base for four shillings and sixpence—about $1.10. As you descend in the grade of practitioners the fees decline also, fillings being inserted for a shilling, and artificial teeth going for a song. The custom prevails of inserting teeth over roots which are unfilled, and, as every one knows, it is a very filthy method.

Our American advertising dentists could learn a thing or two from the sons of Albion, were they in search of such information. The marvellous things they tell in newspapers of their exploits and their own "patent" "soft," "easy-fitting" "cushions" for "tender gums," and the brushes, powders and elixirs which they have in hand, and other allurements for the money, are too numerous to mention. These charlatans are a class by themselves.

The English operating room is not as easily entered as are ours at home, except by the favored few. Our own easy good natnre and carelessness of the feelings of our patients, permits us to open our doors to nearly every caller on the most trivial pretext. They are more careful in this respect. We ought to be.

When one enters a dental goods establishment and asks for anything new, they immediately show something from America. But by persistent questioning and keeping the eyes open, one will finally see a number of inventions and improvements on American instruments which cannot be found in America, because they are contraband. On account of the murky atmosphere in London dentists either have to operate but a few hours daily, or use artificial light. Hence there are many forms of reflectors and globes which we are unaccustomed to see. I found better nerve extractors than we can get at home; likewise syringes, explorers, files, and a number of little odds and ends which have to be picked up here and there as you see them, for, singular to relate, many of my choicest "finds" are not in catalogues or in the advertising pages of any dental journal. In conclusion I have only to state that everywhere I was most courteously received and hospitably entertained, and if I have seen some things to criticise I have been equally unsparing of things and customs at home.—*The Independent Practitioner.*

